# Which Moisturizer to Use in Scar Therapy after Burn Injuries? A Subjective and Objective Skin and Scar Evaluation after Topical Treatment with Dexpanthenol, Aloe Vera, and Plant Oil

**DOI:** 10.3390/medicina59101874

**Published:** 2023-10-21

**Authors:** Mahsa Bagheri, Michelle Werres, Paul C. Fuchs, Harun Seyhan, Rolf Lefering, Gerrit Grieb, Jennifer Lynn Schiefer

**Affiliations:** 1 Clinic for Plastic and Hand Surgery, Burn Care Center, University of Witten/Herdecke, Cologne Merheim Medical Center (CMMC), 51109 Cologne, Germany; 2 Institute for Research in Operative Medicine (IFOM), Faculty of Health, Witten/Herdecke University, 51109 Cologne, Germany; 3 Department of Plastic and Hand Surgery, Burn Center, Medical Faculty, RWTH Aachen University, 52062 Aachen, Germany; 4 Department of Plastic and Hand Surgery, Hospital Havelhohe, 14089 Berlin, Germany

**Keywords:** scar therapy, alhydran^®^, dexpanthenol, teresienoil, tewameter, scar evaluation, burn scar management

## Abstract

*Background and Objectives:* Good scar management in burn care is essential. Nevertheless, there are no consistent recommendations regarding moisturizers for scar management. Our aim was to investigate and compare the effects of commonly used products on normal skin and burn scars. *Materials and Methods:* A total of 30 skin-healthy (control group) and 12 patients with burn scars were included in this study. For an intraindividual comparison, each participant received creams consisting of dexpanthenol (P), aloe vera (A), and a natural plant oil (O) with instructions to apply them daily to a previously defined area for at least 28 days. Objective scar evaluation was performed with Visioscan^®^; Tewameter^®^; Cutometer^®^, and the Oxygen To See^®^ device. Subjective evaluation was performed with an “application” questionnaire, the Patient and Observer Scar Assessment Scale (POSAS), and with the “best of three” questionnaire. *Results:* After (A) a high trend of amelioration of +30%, TEWL was detected on the scar area. Blood flow increased slightly on healthy skin areas after (A) application to +104%. The application of (A) on healthy skin demonstrated a positive effect on the parameters of scaliness (+22%, *p* < 0.001), softness (+14%, *p* = 0.046), roughness R1 (+16%, *p* < 0.001) and R2 (+17%, *p* = 0.000), volume (+22%, *p* < 0.001), and surface area (+7%, *p* < 0.001) within the control group. After (P), a significant improvement of the baseline firmness parameter of +14.7% was detected (*p* = 0.007). (P) also showed a beneficial effect on the parameters of R1 (+7%, *p* = 0.003), R2 (+6%, *p* = 0.001), and volume (+17%, *p* = 0.001). (O) lead to a statistically significant improvement of volume (+15%, *p* = 0.009). Overall, most study participants stated (A) to be the “best of three”. *Conclusions:* (A) performed statistically best, and is a well-tolerated moisturizing product. However, further quantitative studies are needed to provide statistically significant clarification for uniform recommendations for scar therapy.

## 1. Introduction

Scars after burn injuries have a significant impact on the quality of life of burn patients. Thus, scar-management in burn care is one of the central ways to ensure the best possible patient outcome and quality of life.

As scars have an altered skin structure, and therefore alter in various skin functions, there are different approaches to scar-management or therapy. Typically, scarred tissue loses more transepidermal water than healthy skin does. As a response to this increased transepidermal water loss, several inflammatory processes are induced, leading to excessive production of collagen in the skin layers [1,2,3]. This causes reduced elasticity in scars. Another major issue and symptom of scarred tissue is increased itching. Especially in burn patients, often with a high percentage of the total body surface being scarred, the quality of life is significantly reduced by increased itching of burn scars [1,4].

Standard recommendations that burn patients receive as soon as they leave a burn unit include frequent use of moisturizing and lipid-replenishing creams, with an additional mechanical scar massage (or soft tissue mobilization (STM)) [5,6,7]. Here, it has already been shown that when comparing a standard rehabilitation protocol for a burn patient with a scar massage protocol, improvements in pain, itch, scar thickness, melanin, erythema, TEWL, and skin elasticity can be achieved with a combined scar massage protocol [1,8].

An optimal scar cream is necessary not only to massage scars, but also to address further altered skin properties in the scarred tissue. It should ideally be moisturizing, conducive to scar healing, minimally irritating, protective of the skin barrier, and helpful in reducing transepidermal water loss [1].

Currently, there are no specific recommendations regarding moisturizers for scar management after burn injuries. Available recommendations are mostly based on the clinical experience of burn specialists, such as medical staff and employees. Very few are based on scientific evidence [1].

Creams commonly used in the clinical reality, which are also found in the literature, are Alhydran^®^ (Asclepios GmbH, Breisgau, Germany), Panthenol-ratiopharm^®^ (Ratiopharm GmbH, Ulm, Germany) and Theresienöl^®^ (Theresienöl GmbH, Kufstein, Austria). **Alhydran^®^** is an aloe vera-based moisturizer with an occlusive property, and is used for skin hydration and scar aftercare. Aloe vera as the leading ingredient is widely used in natural medicine as a healing agent. Some studies have already shown that aloe vera gels have positive effects, especially in wound therapy [9,10,11]. **Panthenol-ratiopharm^®^** is used as a standard of care in our clinic in the aftercare of burn scars. This is also an occlusive ointment that is supposed to keep transepidermal water loss low. The active ingredient is dexpanthenol being converted to panthotenic acid, a coenzyme constituent which is well-known to have beneficial effects on wound healing and speed up the wound-healing process [12,13]. **Theresienöl^®^**, originating from Austria, consists of purely plant-based ingredients such as apple and lily extract, as well as tocopheryl acetate, also known as vitamin E. In the literature, vitamin E is described to have positive effects on cell repair and epithelialization of injured skin [14,15].

To quantify the success of scar creams, it is necessary to use subjective and objective measuring devices. Subjective evaluation tools have widely been validated in the literature [16,17,18]. Most commonly, the Patient and Observer Scar Assessment Scale (POSAS) and the Vancouver Scare Scale (VSS) are used to evaluate patients’ scar appearance.

Objective scar evaluation tools measure the scar quality with minimal user variability. The assessment includes measurements of components such as firmness, pliability, elasticity, color (pigmentation and erythema), and relief depending on the measurement device [16]. The target parameter elasticity can objectively be measured with the **Cutometer^®^** (Courage + Khazaka Electronics GmbH, Köln, Germany). The measurement principle is based on the elastic properties of the collagenous connective tissue fibers elastin and collagen. The device consists of a probe with which negative pressure is created on the skin. Following it, the depth of penetration of the skin is measured with an optical method that necessitates no physical contact. It reflects the resistance of the skin. After the pressure is released, the skin is simultaneously released again, and it returns to its original state and the elasticity is determined. The behavior of the skin under this measurement method can be graphically represented as a curve, with skin penetration depth plotted against time [19].

Since, physiologically, water continuously evaporates from the body through the skin into the environment, the quality of the skin barrier can be assessed by determining the transepidermal water loss with a **Tewameter^®^** (Courage + Khazaka Electronics GmbH) [20]. There is a hollow cylinder (10 mm in diameter, 20 mm in height) on the probe head of a Tewameter which serves as an open chamber. Inside this hollow cylinder, there are a pair of humidity and temperature sensors that register density gradients generated by evaporation, and transmit them to a microprocessor which evaluates these data. The physical basis of the measuring principle is Fick’s law of diffusion.

Another device is **Oxygen to See (O2C)^®^** (LEA Medizintechnik GmbH, Giessen, Germany), which uses noninvasive laser-Doppler spectrophotometry for measurement of cutaneous and subcutaneous “blood oxygenation (SO_2_)”, “hemoglobin concentration (Hb)”, and “blood flow”. It is achieved by an optical sensor that measures the parameters at a depth of 2 mm to 6 mm. It has been specifically used to objectively quantify the microcirculation of soft tissue and, therefore, the therapeutic success after surgery in several studies [21,22].

**Visioscan^®^** (Courage + Khazaka Electronics GmbH) allows the determination of skin microtopography by using the SELS (surface evaluation of the living skin) parameter [23]. Here, skin smoothness (SEsm), skin roughness (SEr), scaling (SEsc), and wrinkling (SEw) can be calculated. Additionally, the parameters volume, surface area, and the roughness parameters R1 and R2 can be calculated. While the parameters wrinkling, roughness, and flakiness increase with value size, the softness decreases with increasing value.

To establish consistent recommendations for the use of scar creams, it is necessary to obtain quantitative and qualitative evidence concerning different therapeutical strategies, including different moisturizers being used. Our aim was to investigate and compare the effects of three ointment products, Alhydran^®^, Panthenol^®^, and Theresienöl^®^, after consistent treatment on normal skin and on scars after burns, and to establish possible application recommendations.

## 2. Materials and Methods

This monocenter interventional clinical study was approved by the Ethical Review Committee of the University of Witten/Herdecke, Germany (Ethics Review Committee approval number 63/2020), and was conducted from 2020 to 2021 according to the principles of the Declaration of Helsinki.

### 2.1. Scar Group

Patients aged between 21 and 70 years with scar areas after burn injuries were invited to participate in the study at the Burn Center of Cologne Merheim. Here, inclusion criteria were scars after superficial burn injuries, with a minimum area of 10 × 10 cm, or 1% of the total burned surface area (TBSA).

### 2.2. Control Group

For the control group, patients aged between 21 and 68 years with no further skin health issues were invited to participate. Here, exclusion criteria were pathological skin changes and examination of existing scars on the skin areas.

### 2.3. Ointments and Selection of Skin and Scar Areas

Alhydran^®^ (Asclepios GmbH, Germany) is an aloe vera-based gel cream with occlusive properties, and is used for scar aftercare. Panthenol-ratiopharm^®^ (Ratiopharm GmbH, Ulm, Germany), in its pharmaceutical form of a cream, includes the active ingredient of dexpanthenol, which is known to speed up the healing process and to have a beneficial effect on wound healing. Theresienöl^®^ (Theresienöl GmbH, Kufstein, Austria), an oil originating from Austria, consists of purely plant-based ingredients such as apple and lily extracts, as well as tocopheryl acetate, also known as vitamin E.

**Figure 1:**
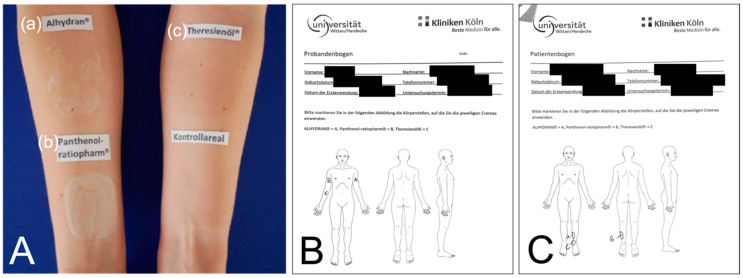
(**A**) Ointment application on the forearms; “Kontrollareal” = control, ointments marked as (**a**–**c**); (**B**) example for marked skin areas for study participant in control group; (**C**) example for marked scar areas for patients in the scar group.

For intraindividual ointment comparison, each study participant was given all three ointment products. All study participants were instructed to apply the ointments once daily to a previously defined skin or scar area for a minimum period of 28 days (see Figure 1). All three areas to treat were marked precisely on a scheme (see Figure 1). The ointments were priorly named as A (Alhydran), B (Panthenol), and C (Theresienoil), and then randomly targeted for the specific areas on the scheme (see Figure 1). Similarly, in the scar group, scar areas of minimum 10 × 10 cm were chosen and randomly targeted to the different ointments.

### 2.4. Skin and Scar Evaluation

The patients of the scar group were examined twice at intervals of four weeks. The first examination took place prior to the ointment application period. After the application period, the defined creamed skin or scar areas of all study participants were examined with objective skin/scar evaluation devices and subjective skin evaluation scores.

For the control group, a single examination appointment was sufficient, in which the creamed skin areas and a control area without application were examined to determine the target variables. For comparison, a nontreated skin area similar to the treated area was examined (see Figure 1).

### 2.5. Objective Scar Measurement Devices

The devices used for the study, manufactured by Courage + Khazaka Electronics GmbH, provided a noninvasive measurement of the required values. The devices included Visioscan^®^ VC 98, Tewameter^®^ TM 300, and Cutometer^®^ dual MPA 580. Furthermore, the Oxygen To See^®^ device from LEA Medizintechnik GmbH was used for the study.

### 2.6. Cutometer^®^

For the evaluation of elasticity, the parameters R0, R2, and R7, mentioned most frequently in the literature, were assessed. The R0 parameter provides information about the firmness of the epidermis. The lower this value, the firmer or less elastic the skin. Since firm skin has a high proportion of connective tissue fibers, it indicates a poor elastic skin condition. Therefore, an ascending value in the progression would be considered positive. The R2 parameter reflects the gross elasticity of the skin, as it indicates the ratio between maximum amplitude in the suction phase and the recovery ability. This value is expressed as a percentage. The closer it is to 1 (= 100%), the more elastic the skin is. An ascending value in the course is to become positive. The R7 parameter indicates how large the proportion of elastic recovery is in the overall curve. The larger this value, the more elastic the skin. Thus, it is to be evaluated positively if the value becomes larger in the course of the study [19].

### 2.7. Tewameter TM 300^®^

Transepidermal water loss is measured with the help of Tewameter TM 300^®^ (in g/h/m^2^. A high TEWL value indicates a high transepidermal water loss. The higher this value, the more impaired the barrier function of the skin. Values above 25 g/h/m^2^ indicate a deteriorating skin condition [20].

### 2.8. Oxygen to See (O2C)^®^

With the aid of this device, the oxygen supply of a perfused tissue can be determined noninvasively. For measurement, the probe is attached to the skin with a transparent and double-sided adhesive tape. This ensures that the probe is applied with a constant pressure and that no pressure artifacts occur during the measurement. The measurable parameters are measured simultaneously. In the context of this work, the relative blood flow was observed above all, since this provided information about the skin blood flow. Since it was a relative parameter, the measurement data were given as a dimensionless quantity (arbitrary units = AU) [24].

### 2.9. Visioscan^®^

Visioscan^®^ consists of a high-resolution camera and computer software called Multi Image Analysis software (Version VS 98), which makes it possible to display the skin topography, or more precisely, the relief of the stratum corneum, in numerical values by following an image analysis procedure. The calculations are based on the distribution of the gray levels of the image. There are 255 gray values. A gray value of 0 means black, and a gray value of 255 means white. The set measurement area during all measurements is 6 × 8 mm. The parameters that can be displayed are skin smoothness (SEsm), skin roughness (SEr), scaling (SEsc), and wrinkling (SEw), also called SELS parameters. Furthermore, the parameters volume, surface area, and the roughness parameters R1 and R2 can be calculated. The volume and surface area parameters provide information about the “smoothness” of the stratum corneum of the skin. The surface parameter shows the ratio between an imaginary “smooth” and the recorded “wavy” surface of the image profile. The volume parameter is calculated from how much virtual fluid would be needed to fill the imaged rectangle within the image capture up to the averaged height of all the mountains present. Thus, the smoother the surface is before “filling”, the less fluid is needed. The roughness parameters roughness depth (R1) and maximum roughness depth (R2) reflect, as the names suggest, the degrees of roughness of the surface. R1 is determined by calculating the distance between the highest peak and lowest valley in the image profile. R2 shows the largest single roughness depth within the profile, which has been divided into five equally sized sections [23].

### 2.10. Subjective Evaluation Tools

Subjective evaluation of the scar appearance in the patient group was carried out with the help of the Patient and Observer Scar Assessment Scale (POSAS) and Application Questionnaires.

### 2.11. POSAS

The POSAS consists of two scales: the Observer Scar Assessment Scale (oPOSAS) and the Patient Scar Assessment Scale (pPOSAS). In the oPOSAS, the investigator answers questions under six main categories, each with two to five subcategories, followed by an overall assessment. Items include circulation (pale, pink, red, purple, mixed), pigmentation (hypo- and hyperpigmentation, mixed), thickness (thicker, thinner), and relief (more, less, mixed). This is followed by the individual pPOSAS, in which the patient indicates his/her subjective perception of each of the characteristics, namely, pain, itching, color, smoothness, thickness, and evenness, as well as another overall assessment. The categories consist of a 10-point scale, with 1 point representing the condition “normal skin” or “no discomfort”, and 10 points denoting “worst scar imaginable” or “most severe discomfort”. Thus, a total score between 6 and 60 points can be achieved.

### 2.12. Application Questionnaire

The participants were asked to complete additional questionnaires at the end of the study. Both the patients and the control group were asked to answer “application questionnaires” to record possible confounding factors that could have a negative effect on compliance or the application method.

To this end, they were asked whether burning, itching, or redness had become noticeable during the application of the ointment preparations, whether an increased scaliness or perspiration had occurred during the application, or whether tension or irritation had become noticeable. In addition, it was asked whether one area of the ointment application was more exposed to the sun than others were, so that a possible disturbing factor could also be recorded here. The overall tolerability for the respective ointments was also evaluated by the study participants. These items were assessed on a Likert scale with values between 0 (= not at all true) and 10 (= totally true).

### 2.13. “Best of Three” Questionnaire

In addition, a questionnaire for direct ointment comparison was evaluated. Here, an investigation of a personal favorite was performed. User-friendliness was addressed by assessing which of the three ointments was easier to spread, absorbed more quickly, subjectively moisturized well, left a better feeling on the skin, and which the patients would be more likely to continue to use.

### 2.14. Statistics

The data were evaluated with MS-Excel^®^ Version 2021and subsequently analyzed and presented by using the statistical program SPSS^®^ Version 26 for Windows^®^. In both study cohorts, skin elasticity and skin hydration were compared intra-individually using a paired *t*-test. The number of patients in the control group was planned considering an α-error of 0.01 for a difference of 2/3 standard deviations. The size of the patient case number in the scar group was based on an α-error of 0.025 to detect a difference of one standard deviation.

## 3. Results

### 3.1. Scar and Control Group

A total of 42 participants were included in this study.

In the scar group, 12 patients with defined scar areas of 10 cm^2^ were included. The mean age of the scar group was 41.8 (range 21–70) years, with four female and eight male patients.

**Figure 2:**
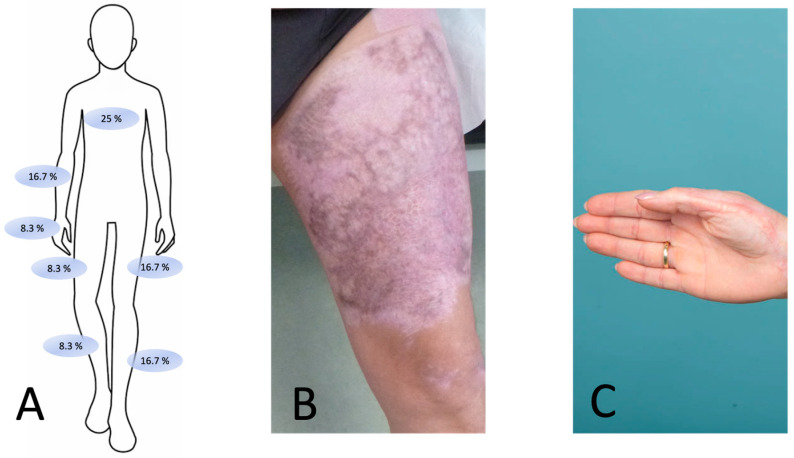
(**A**) Frontal view of the body displaying the treated scar areas as a percentage; (**B**) photograph of a patient with a treated scar area on the left upper leg; (**C**) photograph of a patient with a treated scar area on the thenar area of the right hand.

The examined scar areas are presented graphically in Figure 2. The average age of the scars at the beginning of the intervention was 10 (range 3–15) months.

All of the patients showed a history of second-degree burns, of which 25.0% were superficial-thickness (IIa), 33.3% were partial-thickness (IIb), 33.3% were mixed superficial, and 8.3% were full-thickness burns (III).

The mean duration of the topical application of the ointments was 35 days (range 28–56).

In the control group, a total of 30 participants with no history of large-scale scarring were included.

The mean age was 38.6 (range 24–68) years. Of the control group, 16 were female and 14 were male.

The average duration of ointment application in the control group was 34 days (range: 27–95).

### 3.2. Objective Evaluation

All measurement results of the objective devices are shown in Table A1, Appendix A.

#### 3.2.1. Cutometer^®^

Within the control group, it was shown that the application of the ointment products did not result in a significant improvement in the firmness of the skin (the parameter firmness (R0), see Table A1).

In the scar group, Panthenol^®^ application significantly improved the baseline firmness of 15% (*p* = 0.007).

In both groups, minimal changes from ±2% were detected, with no statistical significance for further parameters, as shown in Table A1.

#### 3.2.2. Tewameter^®^

In the control group, no significant improvement or impairment in transepidermal water loss following the use of the ointment products could be shown (see Table A1).

The scar group showed an average TEWL of 13.51 ± 8.08 g/h/m^2^ for the untreated scar. The application of Alhydran^®^ resulted in a nonsignificant decrease, thus amelioration of TEWL of 30% (*p* = 0.094). The scar areas with Panthenol^®^ application showed only a nonsignificant increase, thus worsening of −2% (*p* = 0.878). After the application of Theresienöl^®^, a nonsignificant decrease, thus amelioration of 9% (*p* = 0.553) could be observed.

#### 3.2.3. O2C^®^

In the control group, the measured flow rate showed a mean value of 27.21 ± 15.02 AU. After the use of Alhydran^®^, the value increased about 104% (*p* = 0.20). With the use of Panthenol^®^, the flow rate increased about 42% (*p* = 0.039), and about 52% (*p* = 0.020) following the use of Theresienöl^®^.

In the scar group an average flow rate of 35.33 ± 25.75 AU at baseline was shown. With Alhydran^®^ application, it increased about 59% (*p* = 0.168). With the application of Panthenol^®^, it decreased about 12% (*p* = 0.927), and with Theresienöl^®^ it decreased about 12% (*p* = 0.844). The changes were nonsignificant, as shown in Table A1.

#### 3.2.4. Visioscan^®^

In the control group, the application of Alhydran^®^ demonstrated a significant positive effect on the parameters of scaliness by (+22%, *p* = 0.000), softness (+14%, *p* = 0.046), roughness depth (+16%, *p* = 0.000), volume (+23%, *p* = 0.000), and surface area (+7%, *p* = 0.000), as shown in Table A1. Panthenol^®^ also showed a beneficial effect on the parameters of roughness depth (+7%, *p* = 0.003), maximum roughness depth (+6%, *p* = 0.001), and volume (+17%, *p* = 0.001). Theresienöl^®^ showed a statistically significant improvement for the parameter volume (+15%, *p* = 0.009).

**Figure 3:**
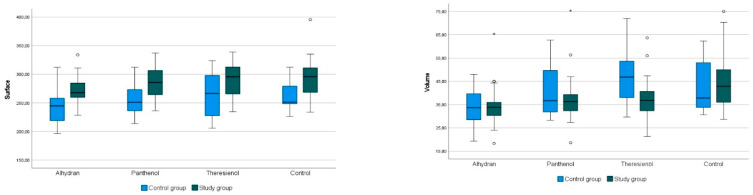
Results of microtopography parameters “surface” and “volume” measured with the Visioscan^®^ device. (* marks statistical significance).

In the scar group, Alhydran^®^, Pathenol^®^, and Theresienöl^®^ application showed no significant changes in the parameters, as presented in Table A1 and Figure 3.

### 3.3. Subjective Evaluation

#### 3.3.1. Application Questionnaire

The survey on ointment usage within the control and scar groups showed that all ointment products were rated as tolerable overall (see Table 1). For a more detailed assessment of tolerability, both the control and scar groups’ participants rated the ointment with the parameters of burning, itching, redness, scaling, and feelings of tension and irritation, each on a scale between 0 and 10, as presented in Table 1.

#### 3.3.2. POSAS

In the Patient Scar Assessment Scale (pPOSAS) of the POSAS, it was observed that there was no significant change in the subjective scar quality in any of the question categories, as presented in Table 2. All categories showed an imperceptible increase in the scores, meaning that the scars did not approach or correspond to a normal skin condition in the course of the study. From a possible total score of 60, the mean total score within the patient collective was 25 (*p* = 0.742).

Similarly, in the Observer Scar Assessment Scale (oPOSAS) of the POSAS, no significant changes could be shown, as presented in Table 3. The overall scar assessment reached a value of 3.25 before application, while it decreased nonsignificantly to 3.00 afterward (*p* = 0.429). The overall assessment showed a lower value during and after treatment of 19.58 (mean = 22.17), without having any statistical significance (*p* = 0.114).

#### 3.3.3. “Best of Three” Questionnaire

In the Table 4 “best of three” patient questionnaire, it was shown that Alhydran^®^ was the overall ointment favorite within the scar group (58%), followed by Panthenol^®^ (25%) and Theresienöl^®^ (17%). Alhydran^®^ scored the highest in four of the six question categories.

## 4. Discussion

One of the key components in the rehabilitation process of burn patients is scar management, especially to ensure that there are no functional limitations and that the quality of life is not affected. Despite the standard recommendations that every burn surgeon provides their patients with, it is qualitative information about moisturizer products that is scarce. The literature does not seem to provide a consistent answer to the simple question as to which specific moisturizer to use in patients’ scar management.

Here, especially with objective evaluation tools, the effect of moisturizers can be measured and compared. To this end, we included 30 skin-healthy patients and 12 patients with mature burn scars in our comparative study. With a mean application period of 35 days, the ointment products Panthenol^®^, Alhydran^®^, and Theresienöl^®^ were applied to skin-healthy and scarred examination areas (see Figure 1), and the effect was objectively and subjectively evaluated afterwards.

In 2017, Klotz et al. showed in a survey of 56 burn units from the Australian, New Zealand, and American Burn Associations that only three respondents were able to provide published citations to support their scar therapy and ointment recommendations [1]. This small number emphasizes the scarcity of evidence-based recommendations in scar therapy.

**Dexpanthenol,** as the leading ingredient of **Panthenol^®^**, has a confirmed moisturizing and skin barrier-enhancing potential [25]. It has also been shown to facilitate wound healing. In almost 80 years of its existence in moisturizing formulas, its role in dermatological conditions is well examined [25].

The use of topical dexpanthenol in scar management, and especially in skin hydration, has shown positive effects. Stettler and colleagues conducted an eight-week-long pilot study with the topical use of a silicone gel containing dexpanthenol and a massage ball on 34 subjects with hypertrophic scars. Here, skin hydration measured by corniometry increased significantly by 25%, and TEWL decreased by 7% during the study period, indicating an increased strength of skin barrier [25,26]. In another randomized double-blind placebo-controlled study, two topical dexpanthenol formulations were assessed on epidermal barrier function [27,28]. Here, after seven days of dexpanthenol treatment, hydration significantly increased, with a significantly decreased TEWL. This finding supports the favorable impact of dexpanthenol on proactive scar management, thus preventing hyperproliferation of scars by enhancing adequate skin repair in superficial wound healing, retaining its hydrating properties [27,29].

Despite these favorable outcomes, we were not able to show any significant changes in TEWL in both the scar and control groups (see Table A1). However, when analyzing the evolution of elasticity before and after treatment with the Cutometer^®^ device, we were able to show a significant reduction of 15% of the firmness parameter (R0) (*p* = 0.007) in the scar group after Panthenol^®^ treatment. All other elasticity-related parameters considered for this work did not significantly change, not even for skin-healthy patients. In skin-healthy patients, this could suggest that measurable improvements in elasticity may not be achievable, contrary to medial advertisements.

Underlining the hydration effects of Panthenol^®^, we were able to show, in terms of skin microtopography, positive significant changes for the roughness parameters R1 (+6%) and R2 (+8%), and for the volume parameter (+17%).

Furthermore, it has been well described that with the use of dexpanthenol on scars, pain and itching scores can decrease from baseline assessments [26], which is in line with our results (see Table A1). Similarly, in the subjective evaluation, we were able to show that there was no pain in 100%, and no itching in 90% of people in the scar group (see Table 1).

Recommendations concerning topical use of scar gels containing silicone already exist in the literature, especially for burn patient groups, which are to benefit from early use on nonmatured scars [30]. Still, it is the quantitative information about the effects of dexpanthenol-formula on scars which is missing. Several studies on postprocedural wound healing exist in the literature, confirming that dexpanthenol upregulates genes that are critical for wound healing [12], which makes it especially necessary in postprocedural wound healing. Furthermore, new insights into wound healing suggest that the traditional three phases of wound healing (inflammation, proliferation, and remodeling) occur in an overlapping or even a simultaneous manner, rather than successively, necessitating a clinical approach targeting the phases simultaneously. Several in vivo and in vitro studies have shown that dexpanthenol may assist wound healing in all of the three stages through modulation of inflammation, support of cell proliferation, and protection from infection and free-radical damage [27].

**Alhydran^®^** is a medical moisture retention cream (MMRC), which is a type of oil in water emulsion with freshly processed pure Aruba aloe vera gel, oils, and other fatty ingredients. With its aloe vera component, it is described to have a moisturizing effect combined with an occlusion effect through the fatty acids [31].

The minimization of TEWL seems to be one of the most important approaches to scar treatment, as with the minimization, hypertrophic scarring can be prevented [1,8]. An increased production of collagen due to disrupted skin barrier functions in scarred tissue leads to decreased scar elasticity [2,3]. Thus, the main goal is to bring the TEWL close to levels of normal skin through a balanced degree of occlusion and hydration [31].

With regard to this parameter, no significant change in the TEWL value was observed in our study, either in the control group or in the patient collective, using the ointment products tested (see Table A1).

Nevertheless, interestingly, after the application of Alhydran^®^, there was a nonsignificant, but more than 30-fold (vis-à-vis Panthenol^®^) or three-fold (vis-à-vis Theresienöl^®^) improvement in TEWL in the scar areas of the control group. In the skin-healthy control group, a tendency to deterioration of −4 to −11% was observed for the products Alhydran^®^ and Theresienöl^®^. A possible explanation for a deterioration in TEWL despite ointment application might be a change in environmental exposure, such as exposure to sun or dry air. Another possible reason for inconclusive results when examining scar areas with the Tewameter^®^ is that the device cannot guarantee uniform measurements in the entire scar area [32].

In contrast to our results, the authors Moortgat et al. were able to show a significant improvement in skin hydration after the use of Alhydran^®^ on normal skin in their pilot study. Here, skin hydration was measured 30 min after application by using a Corneometer^®^ (Courage+Khazaka Electronic GmbH, Köln, Germany). Similar to our results, no such effects were reproducible in the scar areas, presumably due to impaired skin barrier functions, and thus possibly absorption capacity [33].

The occlusion ability of Alhydran^®^ in comparison with silicone gels and silicone overlays was well demonstrated in a study by Hoeksema et al. [32,34]. Methodically, the authors performed tape stripping as a comparison to normal skin functioning as an imitation scar to mimic the two main characteristics, which are water loss and a thinned stratum corneum. They measured the TEWL with the Tewameter^®^ and the hydration state of the stratum corneum compared with intact skin and scar-like control over a 3–4 h period. Here, Alhydran^®^, as a hydrating gel cream, was shown to be equally occlusive as a silicone gel, both reducing the TEWL significantly.

Thus, in scar or wound therapy, it might be of advantage to use a cream as opposed to silicone overlays in order to avoid adverse events, such as maceration of the skin, that can occur under silicon overlays. This would be entirely in the spirit of a balanced scar treatment [32,34].

This is also in line with the subjective evaluation results of our study. The results of the “application questionnaire” reflect the moisturizing quality of Alhydran^®^. Smoothness was present in over 60% of people in the study and the control group after application. Scaliness was absent in over 80% of participants of either group.

Aside from the hydrating effect, we observed interesting effects concerning blood flow evolution after Alhydran^®^ application. Although when examining the skin healthy areas and the scars with the O2C^®^ device no significant changes in blood flow values were observed under any of the ointment products tested, the greatest increase in blood flow was seen after the use of Alhydran^®^, with +104% on healthy, scar-free skin of the control group. On scarred skin, Alhydran^®^ showed the best increase in blood flow of 60%, while Panthenol^®^ and Theresienöl^®^ showed a decrease of up to 12% (see Table A1). This might underline the positive effects of Alhydran^®^ on scar maturation, providing a possible increase of vascularization, and thus minimization of excessive scarring.

When evaluating the skin microtopography with the Visioscan^®^, the highest significant changes were seen in the control group after Alhydran^®^ application. Here, significant positive effects on the parameters scaling (+22%), R1 (+16%), R2 (+18%), and the smoothness parameters volume (+23%) and surface area (+7%) were detected. Compared to Panthenol^®^, only one-third of the effect was seen for the roughness parameters R1 and R2. The results of a study by Rondas et al. in 2017 are similar, where 17 of 18 patients showed significant clinical and subjective improvement in the main examined skin symptoms (dryness, itching, scaling) for venous eczema after using Alhydran^®^ for four weeks [31]. However, it should be noted that their study was conducted for clinical evaluation without the use of scales or objective measurement devices.

To comprehensively evaluate the moisturizing effects of the ointments on scars and determine their potential to significantly impact this critical parameter, a larger cohort of scar patients is essential. This investigation is crucial, as transepidermal water loss (TEWL) stands as a foundational factor in scar progression and deformity.

The Austrian **Theresienöl^®^** is a natural product consisting of herbal ingredients, whose formula has remained unchanged for the last 700 years [35]. In our study, no significant changes of normal skin or scar parameters were detected after the application of Theresienöl^®^. Nevertheless, in the literature, many beneficial properties are attributed to the product.

Theresienöl^®^ contains Butyrumbovis as a carrier of all active ingredients such as tocopherol and tocopherol acetate (vitamin E). They have positive effects on cell repair and epithelialization of injured skin. An antioxidant effect of the ointment is provided by the fruit extract from Pyrusmalus, which is rich in polyphenols. Furthermore, tannic and salicylic acids are responsible for the skin pH. Antifungal and antibacterial effects are further provided by the leaf extract from Stellrioides. In the literature, it is described that this combination of anti-inflammatory and antipruritic effects is meant to promote an efficient healing process [14,15].

The human skin consists of both water- and lipid-soluble compartments containing bioactive antioxidants. Many of them, such as vitamins C and E, cannot be produced endogenously, and must be provided externally [36]. Both active ingredients are used synergistically in the cosmetics industry. The antiaging effect of vitamin C as an antioxidant and mediator of light damage and melanogenesis can reach deeper layers of the skin through vitamin E. In addition, vitamin E is supposed to reduce hyperpigmentation, in particular. In a monocenter intraindividual split-face comparison study, the effects of a vitamins E and C serum were evaluated on 50 women. Here, skin color, elasticity, and radiance significantly improved. Measurements were taken with objective evaluation devices such as a Mexameter^®^, Cutometer^®^, Tewameter^®^, and more [36].

Although Theresienöl^®^ is also described to enhance blood supply [35], quantitatively in our results, no increase of blood flow could be shown with the O2C^®^ device. Subjectively, 90% of the participants described no redness after the use of the ointment, as shown in Table 1.

In a prospective multicenter study of 53 deeper superficial burn injuries in the face, the authors postulate that Theresienöl ^®^ penetrates the burn eschars, while not separating them from the wound, allowing good epithelialization subcrustal. Nevertheless, in the presented study, the authors describe a good penetration of Theresienöl^®^ into the eschars compared to “other” products, while not further differentiating the “other” products [35].

In another monocenter prospective study, the effect of Theresienöl was examined on 17 patients with vulvar leukoplakia. A significant reduction of itching was noted, which was similar to our results, where both in the study and control groups there was a complete absence of itching after Theresienöl^®^ application (see Table 1). Still, a limitation of the mentioned study is the evaluation of the treated areas with only a visual analogue scale (VAS) [14].

In another prospective multicenter cohort study, the therapy-refractory skin injuries and also burns of 1354 patients were treated with Theresienöl^®^. Here, similar positive effects of Theresienöl^®^ were shown. An 89% improvement rate in inflammation, 88% reduction in pruritus, 87% improvement in epithelialization, and a 91% improvement rate for wound closure were shown. Despite these favorable results, evaluation was only made visually by VAS, and that did not include the control group [15].

Data concerning the wound healing and analgetic properties of Theresienöl^®^ exist in the literature [35,37], but to the best of our knowledge, there is paucity of information about the effect of scar management on healing of wounds.

Some limitations of the study design must be noted. Although all the measurements were taken by the same examiner, inaccuracies might still be present. The major part of the measurements was taken in the European summer months. Here, attempts were made to ensure that the study rooms corresponded to the conditions obtained at the respective measurement times (e.g., to ensure minimal sweating). Given that the measurements were also taken on potentially sun-exposed skin and scar areas, it might have led to detrimental effects on skin moisture and overall skin condition.

Overall, the results in our study concerning subjective evaluation show that patients in both the study and cohort groups tolerated all three products well (see Table 1, Table 2, Table 3 and Table 4).

In the classic questionnaires PSAS and OSAS, the subjective evaluation of the patients and the observer hardly showed any noticeable changes in the scar-management rating after ointment application. Nevertheless, according to our results, the scar evaluation of the patient collective tends to perform worse than that of the examiner. An improvement in the subjective perception of the scar quality triggered by the ointments could not be shown in our study.

In the “best of three” questionnaire of this study, Alhydran^®^ clearly wins. However, there is still a lack of high-quality studies that allow more precise statements, especially regarding comparability [34].

## 5. Conclusions

Skin moisturizers constitute the central pillar of scar management and therapy. A uniform recommendation for one specific moisturizer or quantitative data concerning its effects is scarce in the literature. This study sought to show significant changes in skin evaluation parameters after the consistent use of Alhydran^®^, Panthenol^®^, and Theresienöl^®^ in healthy skin areas and scars. Specifically, Alhydran^®^ performed statistically best, and is a well-tolerated moisturizing product.

Fortunately, it seems that in healthy skin areas, an improvement of the examined parameters was not possible. However, to provide statistically significant clarification of the trends already identified in our study, especially with the aim of providing consistent and uniform recommendations for an ointment product for scar therapy, further quantitative studies are needed.

## Figures and Tables

**Table 1:** Results of the application questionnaire.

	Control Group			Scar Group		
**Parameter**	(A)	(P)	(O)	(A)	(P)	(O)
**Pain**	10.0%	6.7%	0%	8.3%	0%	0%
**Itching**	0%	3.3%	0%	16.7%	8.3%	0%
**Smoothness**	63.3%	53.3%	56.7%	58.3%	58.3%	58.3%
**Perspiration**	10.0%	6.7%	6.7%	0%	8.3%	0%
**Redness**	3.3%	3.3%	3.3%	0%	8.3%	8.3%
**Scaliness**	0%	0%	0%	16.7%	8.3%	16.7%
**Tension sensation**	3.3%	3.3%	0%	16.7%	33.3%	16.7%
**Irritation**	6.7%	3.3%	3.3%	0%	8.3%	8.3%
**UV-exposure**	50%	50%	50%	8.3%	8.3%	16.7%
**Tolerability**	96.7%	93.3%	93.3%	100%	100%	100%

**Table 2:** Results of the pPOSAS of the scar group.

pPOSAS		Before	After	*p*-Value
**Pain**	mean	1.50	1.58	0.674
	SD	1.168	1.240	
**Itching**	mean	1.67	1.83	0.809
	SD	1.371	1.749	
**Color**	mean	4.33	4.25	0.900
	SD	2.103	1.288	
**Smoothness**	mean	4.25	4.33	0.912
	SD	2.491	2.570	
**Thickness**	mean	2.92	3.83	0.403
	SD	2.575	2.082	
**Evenness**	mean	4.42	4.92	0.491
	SD	2.503	2.610	
**Overall Score**	mean	4.75	4.25	0.491
	SD	2.050	2.417	
**Total Score**	mean	23.83	25.00	0.724

**Table 3:** Results of the oPOSAS of the scar group.

oPOSAS		Before	After	*p*-Value
**Circulation**	mean	2.42	2.67	0.536
	SD	1.165	1.435	
**Pigmentation**	mean	3.50	2.92	0.131
	SD	1.314	0.900	
**Thickness**	mean	3.58	2.75	0.085
	SD	1.621	0.754	
**Relief**	mean	3.50	2.83	0.151
	SD	1.446	0.937	
**Flexibility**	mean	3.08	2.83	0.339
	SD	1.676	1.193	

**Table 4:** Results of the “best of three” questionnaire of the scar group.

	Alhydran	Panthenol	Theresienöl
**Best distributability**	33.3%	0%	66.7%
**Fastest absorption**	58.3%	0%	41.7%
**Best moisturization**	33.3%	50%	16.7%
**Best skin feeling**	58.3%	25%	16.7%
**Continuing to use**	58.3%	25%	16.7%
**Personal favourite**	58.3%	25%	16.7%

## Appendix A

**Table A1:** Results of the objective skin and scar evaluation of the skin and scar areas before and after treatment with the ointment products Alhydran^®^ (A), Panthenol^®^ (P), and Theresienöl^®^ (O).

	Skin b.T.	(A)	(P)	(O)	Scar b.T.	(A)	(P)	(O)
**Cutometer^®^**								
**R0**								
Mean	1.104	1.122	1.186	1.135	0.746	0.762	0.856	0.769
SD	0.228	0.175	0.299	0.287	0.335	0.408	0.361	0.329
**Amelioration in %**	-	+1.63	+7.43	+2.81	-	+2.1	+14.7	+3.08
***t*** **-test**	-	0.666	0.034	0.506	-	0.803	0.007	0.647
**SD**	0.041	0.032	0.055	0.052	0.097	0.118	0.104	0.095
**R2**								
**Mean**	0.864	0.873	0.857	0.866	0.812	0.800	0.843	0.793
**SD**	0.111	0.083	0.102	0.096	0.091	0.103	0.073	0.104
Amelioration in %	-	+1.04	−0.81	+0.23	-	−1.5	+3.8	−2.3
*t*-test	-	0.510	0.507	0.882	-	0.532	0.128	0.559
SD	0.020	0.015	0.019	0.018	0.026	0.030	0.021	0.030
**R7**								
**Mean**	0.585	0.577	0.577	0.572	0.532	0.535	0.560	0.516
**SD**	0.152	0.126	0.150	0.143	0.109	0.122	0.100	0.130
**Amelioration in %**	-	+1.37	+1.37	+2.22	-	+0.6	+5.3	−3.0
***t*** **-test**	-	0.615	0.429	0.454	-	0.876	0.272	0.607
**SD**	0.028	0.023	0.028	0.026	0.031	0.035	0.029	0.037
**Tewameter^®^**								
**TEWL**								
**Mean**	15.87	16.48	14.35	17.60	13.51	9.45	13.80	12.30
**SD**	9.21	10.10	8.34	14.24	8.08	4.10	6.18	4.31
**Amelioration in %**	-	−3.77	+8.8	−10.7	-	+30.37	−2.2	+8.9
***t*** **-test**	-	0.735	0.205	0.487	-	0.094	0.878	0.553
**SD**	1.68	1.84	1.52	2.60	2.332	1.184	1.784	1.244
**O2C^®^**								
**Flow**								
**Mean**	27.21	55.62	38.76	41.10	35.33	59.58	34.58	33.50
**SD**	15.02	62.21	31.05	32.06	25.75	65.82	29.43	29.90
**Amelioration in %**	-	+104.4	+42.4	+51.0	-	+59.3	−12.2	−11.8
***t*** **-test**	-	0.020	0.039	0.020	-	0.168	0.927	0.844
**SD**	2.79	11.55	5.77	5.95	7.43	19.0	8.50	8.63
**Visioscan^®^**								
**S_er_ (roughness)**								
**Mean**	2.22	1.99	2.13	2.24	3.85	3.04	2.58	3.16
**SD**	1.92	1.25	1.20	1.67	4.25	2.96	1.76	2.24
**Amelioration in %**	-	10.36	4.05	−0.9	-	11.7	33.0	17.9
***t*** **-test**	-	0.491	0.671	0.953	-	0.188	0.325	0.461
**SD**	0.35	0.23	0.22	0.30	1.23	0.86	0.51	0.65
**S_esc_ (Scaling)**								
**Mean**	0.49	0.38	0.43	0.46	0.70	0.49	0.58	0.75
**SD**	0.17	0.13	0.17	0.12	0.46	0.20	0.26	0.42
**Amelioration in %**	-	22.45	12.24	6.12	-	30.0	17.1	−7.1
***t*** **-test**	-	0.000	0.053	0.308	-	0.074	0.255	0.698
**SD**	0.03	0.02	0.03	0.02	0.13	0.06	0.08	0.12
**S_esm_ (Smoothness)**								
**Mean**	111.90	96.25	106.70	104.31	158.39	171.35	184.54	196.04
**SD**	48.62	30.84	46.55	35.24	58.15	103.03	103.88	100.29
**Amelioration in %**	-	13.99	4.65	6.80	-	−8.2	−16.5	−23.8
***t*** **-test**	-	0.046	0.316	0.376	-	0.517	0.332	0.097
**SD**	8.88	5.63	8.50	6.43	16.79	29.74	29.99	28.95
**S_ew_ (wrinkling)**								
**Mean**	74.28	74.72	73.04	69.65	152.04	173.02	143.42	158.11
**SD**	32.29	22.32	29.12	26.99	69.46	121.24	68.24	71.36
Amelioration in %	-	−0.60	1.67	6.23	-	−13.8	5.7	−3.99
*t*-test	-	0.929	0.726	0.397	-	0.405	0.480	0.703
SD	5.90	4.08	5.32	4.93	20.05	35.00	19.70	20.60
**R1**								
**Mean**	45.4	38.1	42.7	43.3	42.89	39.39	44.72	48.50
**SD**	8.60	6.33	8.83	5.46	8.20	12.31	13.29	10.22
**Amelioration in %**	-	16.08	5.95	4.63	-	8.2	−4.3	−13.1
***t*** **-test**	-	0.000	0.003	0.185	-	0.242	0.633	0.052
**SD**	1.57	1.16	1.61	1.00	2.37	3.55	3.84	2.95
**R2**								
**Mean**	38.41	31.64	35.82	36.39	34.00	31.08	36.22	39.14
**SD**	7.23	5.12	7.65	4.23	7.05	10.46	9.60	8.11
**Amelioration in %**	-	17.63	6.74	5.26	-	8.6	−6.5	−15.1
***t*** **-test**	-	0.000	0.001	0.146	-	0.306	0.418	0.044
**SD**	1.38	0.93	1.40	0.78	2.04	3.02	2.77	2.34
**Volume**								
**Mean**	44.82	34.56	37.37	38.29	42.25	33.81	40.44	47.56
**SD**	11.38	5.24	10.05	8.04	10.73	8.05	11.29	12.25
**Amelioration in %**	-	22.90	16.62	14.57	-	20.0	4.3	−12.6
***t*** **-test**	-	0.000	0.001	0.009	-	0.069	0.693	0.291
**SD**	2.08	1.51	1.83	1.47	3.10	2.32	3.26	3.54
**Surface**								
**Mean**	292.19	271.57	286.31	291.38	263.25	244.40	257.34	263.41
**SD**	33.41	23.38	26.46	27.50	26.24	33.12	30.87	39.61
**Amelioration in %**	-	7.03	2.01	0.62	-	7.2	2.2	−0.1
***t*** **-test**	-	0.000	0.174	0.886	-	0.031	0.442	0.986
SD	6.10	4.27	4.83	5.02	7.57	9.56	8.91	11.43

Statistical significance was considered when *p* < 0.01 in the control group and *p* < 0.025 in the scar group. Skin b.T.: examined healthy skin area of the control group before treatment; scar b.T.: examined scar area of the scar group before treatment.
